# COPD diagnosis related to different guidelines and spirometry techniques

**DOI:** 10.1186/1465-9921-8-89

**Published:** 2007-12-04

**Authors:** Lennart Nathell, Madelene Nathell, Per Malmberg, Kjell Larsson

**Affiliations:** 1 Personal Injury Prevention Section, Department of Clinical Neuroscience, Karolinska Institutet, Stockholm, Sweden; 2 Medical advisor, Deparment of Medical Affairs, Boehringer Ingelheim, Stockholm; 3 National Institute for Working Life, Stockholm, Sweden; 4 National Institute of Environmental Medicine, Karolinska Institutet, Stockholm, Sweden

## Abstract

The aim was to compare the diagnosis of COPD among smokers according to different international guidelines and to compare the outcome when using slow (SVC) and forced vital capacity (FVC).

In order to find current smokers a questionnaire was sent to persons who had been on sick leave for more than two weeks. Those who smoked more than 8 cigarettes per day were invited to perform a spirometry.

Totally 3,887 spirometries were performed. In this sample 10.2% fulfilled the NICE COPD-criteria, 14.0% the GOLD COPD-criteria and 21.7% the ERS COPD criteria. The diagnosis according to NICE and GOLD guidelines is based on FVC and in the ERS guidelines the best value of either SVC or FVC is used. Thus, substantially more subjects with COPD were found when the best of either SVC or FVC was used. Forced VC tended to be higher than SVC when lung function was normal and in those with mild obstruction prior to bronchodilatation whereas SVC exceeded FVC after bronchodilatation in those who had severe bronchial obstruction.

The diagnosis of COPD is highly depending on which guidelines are used for defining the disease. If FVC and not the best of SVC and FVC is used when defining COPD the diagnosis will be missed in a substantial number of patients.

## Background

Chronic obstructive pulmonary disease (COPD) is one of the leading causes of morbidity and mortality amongst the adult population worldwide [1]. Spirometry is the gold standard for diagnosing and monitoring progression of COPD [2] which is defined by irreversible lung function impairment with a reduced FEV_1_/vital capacity (VC) ratio. However, differences in the definition of COPD in guidelines and consensus statements make it difficult to quantify the morbidity and to make comparisons between countries. In addition, there are different recommendations in the major guidelines and consensus statements concerning how to perform spirometry [2-4].

In the present study smokers were identified among patients being on sick leave registered in a Swedish database (Collective Bargaining Goup Sickness Insurance). The smokers were invited to perform a spirometry and the aim of the study was to determine the prevalence of COPD using the definitions and recommendations from the European Respiratory Society (ERS) from 1995, the National Institute for Clinical Excellence (NICE) guidelines and the National Heart, Lung, and Blood Institute (NHLBI)/World Health Organization (WHO) Global Initiative for Chronic Obstructive Lung Disease (GOLD). A further aim was to compare the number of subjects with COPD when the COPD diagnosis was based on slow (SVC) or forced vital capacity (FVC) when defining airway obstruction (FEV_1_/VC).

## Methods

In order to identify current smokers for a smoking cessation programme a questionnaire containing questions on smoking habits was sent to persons 40 to 60 years of age, who had been on sick leave, regardless of cause, for more than two weeks. The persons were identified using the database from the Collective Bargaining Goup Sickness Insurance, AGS (in Swedish: Avtalsgruppsjukförsäkring) [5]. The questionnaire was sent to all persons registered in AGS during the period 1 April 1998 to 30 November 2000. To find persons with a potential risk of having COPD those who, according to the questionnaire, currently smoked more than eight cigarettes per day were invited to perform a spirometry.

Lung function testing was performed at ten different laboratories by experienced and specially trained technicians. Regular meetings were held to reinforce the recommended techniques. Spirometry was performed according to the ATS recommendations [6] with a few modifications. The spirometry was performed in the sitting position and a nose clip was used. After 2 – 3 slow expiratory vital capacity measurements, at least three forced expirations were performed. Spirometry was performed before and, in a selected group of patients, 15 minutes after inhalation of salbutamol dry powder (0.8 mg Ventolin™ Discus™, GlaxoWellcome). Reversibility test was only performed in those who, prior to bronchodilatation, had a SVC/FEV_1 _or FVC/FEV_1 _below 0.75. Short acting bronchodilator medication was withheld four hours and long acting bronchodilators twelve hours before the reversibility testing. European reference values were used [7].

### Definitions

The definitions and recommendations for defining COPD from the ERS consensus statement from 1995 [4], the NICE guideline [3], and the GOLD guidelines [2] were used to calculate the prevalence of COPD (table 1). In the NICE guidelines a FEV_1 _< 80% of predicted value is required for a COPD diagnosis. To fulfil the COPD definition according to the ERS guidelines FEV_1_/VC has to be < 88% (men) or < 89% (women) of predicted value whereas a FEV1/VC ratio < 0.7 is required in NICE and GOLD recommendations. InNICE and GOLD guidelines only FVC is used while the best of FVC and SVC is used in the ERS recommendations. The ATS/ERS standards published in 2004 [2] are identical to the GOLD guidelines in this context and are therefore not specifically considered.

**Table 1:** Definitions of COPD according to the ERS consensus statement, the NICE and the GOLD guidelines.

	**FEV_1_/(VC or FVC)**	**FEV1**	**Remarks**
**ERS**	< 88% pred for men	Only for staging	
	< 89% pred for women		
**NICE**	< 70% absolute value	< 80% predicted	
**GOLD**	< 70% absolute value	Only for staging	Post-bronchodilator values

In the present study lung function was calculated after bronchodilatation, according to NICE, ERS and GOLD guidelines and FEV_1_/VC ratio was calculated by using only FVC or the best out of FVC and SVC. The study was approved by the Ethics Committee at Karolinska Institutet, Stockholm (reg.no. 98:044).

## Results

**Figure 1:**
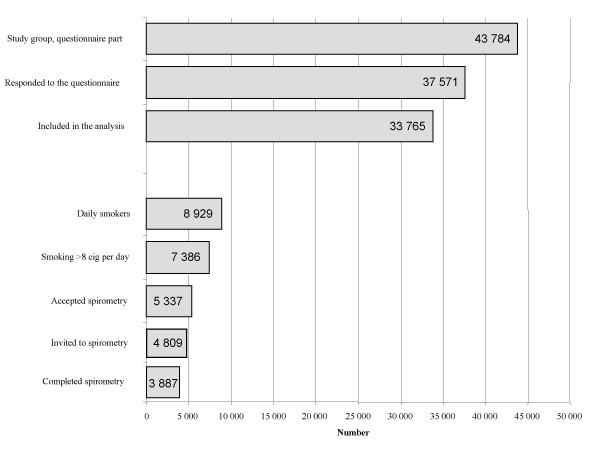
Number of participants in each step of the study.

During the study period 46,734 sick leave periods were registered in the AGS in the selected geographical areas for persons 40 to 60 years of age. Of these 2,841 were multiple sick leave periods, 47 persons were deceased and 62 persons had either unknown or secret addresses. The questionnaire was sent to the remaining 43,784 subjects. The initial response rate was 55% and after two reminders the response rate was 86%. Of the 37,571 responses, 90% (33,765) were complete and of those daily smoking was reported by 26% (8,929) of whom 83% (7,386) smoked more than 8 cigarettes per day. Of those, 5,337 accepted to undergo lung function testing. For different reasons (mental disorder, n = 363, malignant tumor, n = 87, abuse of alcohol or drugs, n = 40, could not speak, write or understand Swedish, n = 22, other complicated illness, such as recent myocardial infarction, n = 16) 528 patients were excluded from the lung function testing. Of the 4,809 subjects invited to spirometry 3,887 completed the examination. The reasons for not participating were: already stopped smoking (n = 22), other illness such as hernia, facial paralysis, low back pain, alcohol abuse (n = 21), moved from the area (n = 8), deceased (n = 4), not acceptable technique (n = 4), lost interest or not stating a reason (n = 863). The selection procedure is shown in (figure 1).

Of those who completed spirometry 1,763 (45.4%) were men, 51.6 (46.2 – 56.1) years of age (median and 25^th ^-75^th ^percentiles), and 2124 (54.6%) were women, 50.8 (46.2 – 56.1) years of age.

In this group of 3887 subjects who smoked more than 8 cigarettes per day 10.2% had COPD according to NICE criteria and 14.0% had COPD according to GOLD criteria. According to these criteria only FVC is used to calculate FEV_1_/FVC ratio. This means that 3.8% (148 individuals) with mild COPD were identified according to the GOLD guidelines but missed when the the NICE-guidelines, i e when the diagnosis of COPD requires FEV_1_< 80% of predicted value, were used. When using the ERS recommendations, using best value of SVC or FVC when calculating FEV_1_/VC ratio the prevalence of COPD was 21.7% (table 2).

**Table 2:** Diagnosis of COPD and reversibility tests.

	**ERS**	**NICE**	**GOLD**
COPD diagnosis using only FVC (%)	17.6	**10.2**	**14.0**
COPD diagnosis using best value of SVC and FVC (%)	**21.7**	11.7	16.8

**Reversibility among subjects with COPD **(according to ERS, NICE and GOLD guidelines using the best of SVC and FVC)			
N	861	446	652
Percent of predicted (mean; 95%CI)	4.8 (4.4–5.1)	5.5 (5.0–6.0)	3.7 (3.3–4.2)
Percent of pre-bronchodilator (mean; 95%CI)	6.9 (6.3–7.5)	9.2 (8.3–10.1)	6.0 (5.3–6.7)

COPD according to the NICE, GOLD and ERS guidelines in 3887 smokers who were smoking more than 8 cigarettes per day at the time of the trial. The prevalence has been calculated using either FVC or the best of FVC and SVC. Figures in bold indicate the analysis which is recommended in the guidelines, respectively. The table is also showing reversibility after inhalation of 0.8 mg of salbutamol as percent of predicted value and as percent of pre-bronchodilator value.

**Figure 2:**
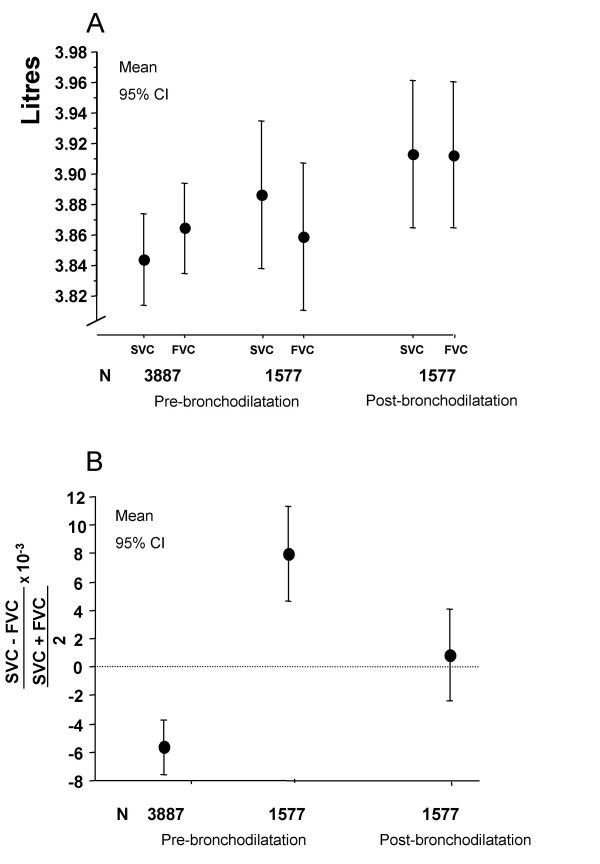
**A. **Slow (SVC) and forced vital capacity (FVC) before bronchodilatation in 3887 smokers and pre- and post-bronchodilatation in 1577 smokers with a pre-bronchodilator FEV_1_/VC-ratio below 0.75. **B. **The difference between SVC and FVC corrected for VC-level in the same smokers as in panel A.

**Figure 3:**
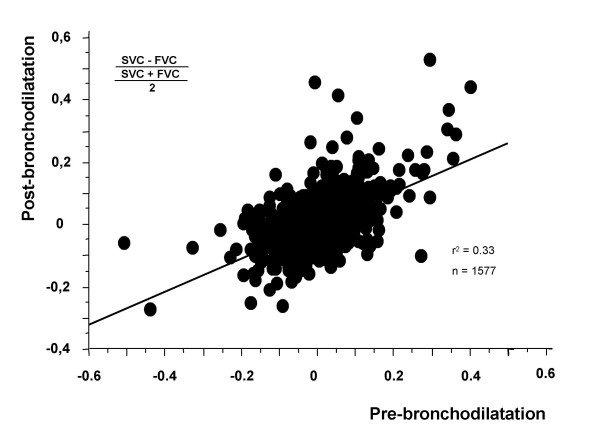
Relationship between pre- and post-bronchodilator slow (SVC) and forced (FVC) vital capacity corrected for VC-level in 1577 smokers with a pre-bronchodilator FEV_1_/VC -ratio below 0.75.

**Figure 4:**
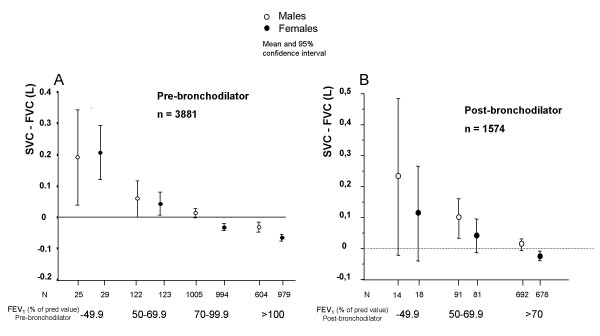
**A. **Difference between pre-bronchodilator slow (SVC) and forced (FVC) vital capacity related to FEV_1 _as percent of predicted value prior to bronchodilatation in 3881 smokers. Due to difficulties in taking instructions or technical errors data from 6 spirometries were not included in the analyses which thus are based on 3881 and not 3887 smokers. **B. **Difference between post-bronchodilator slow (SVC) and forced (FVC) vital capacity related to FEV_1 _as percent of predicted value after bronchodilatation in 1574 smokers with a pre-bronchodilator FEV_1_/VC -ratio below 0.75. Due to unacceptable measurements or technical errors data from 3 spirometries were not included in the analyses which thus is based on 1574 and not 1577 smokers.

Prior to bronchodilatation FVC was higher than SVC in the total groups of smokers (n = 3887) whereas the opposite was the case in those 1577 subjects who had a pre-bronchodilator FEV_1_/VC-ratio below 0.75 (figure 2). Bronchodilatation abolished this difference (figure 2). There was a fair, but not excellent, correlation (r = 0.57) between the difference between SVC and FVC corrected for the VC level when pre- and post-bronchodilator values were compared (figure 3). Pre-bronchodilator SVC was higher than FVC in those with the lowest pre-bronchodilator FEV_1 _while the opposite was found in those with normal FEV_1 _(figure 4A). This was obvious prior to bronchodilatation (total group) but remained when analyses of post-bronchodilator values were analysed in those with a pre-bronchodilator FEV_1_/VC ratio below 0.75 (figure 4B).

## Discussion

In the present study of almost 4000 smokers, smoking more than 8 cigarettes per day, it was demonstrated that the prevalence of COPD differed depending on which guidelines are used and whether the best of slow and forced VC or only FVC were used. It was also shown that FVC exceeded SVC in persons with normal lung function as assessed by spirometry wheras the opposite was found in patients with impaired lung function. Inhalation of a bronchodilator attenuated, but did not abolish, the effect of lung function impairment on the difference between SVC and FVC.

The definition of COPD is arbitrary and varies between different consensus statements and guidelines. In the present study the number of smokers, 40–60 years of age, who got COPD diagnosis varied with a factor two (from 10% to 22%) depending on what definition was used. This variation exceeds somewhat was recently was demonstrated in an epidemiological setting which, based on a random sample of 666 subjects, demonstrated prevalence figures of 7.6 – 14.1% when defining COPD according to different recommendations. [8]. The COPD diagnosis was thus highly dependent on which guidelines the diagnosis was based but also on the measurement of vital capacity. If only FVC was measured the prevalence of COPD was up to 4.1 percentage units lower than if a slow SVC also was measured and the best value of SVC and FVC was chosen for the FEV_1_/vital capacity ratio.

The main reason for the large difference in the prevalence of COPD between the ERS and NICE definitions is that the NICE guidelines require a FEV_1 _less than 80% of the predicted value. In the GOLD and the ERS/ATS guidelines from 2004 post-bronchodilator values are used for calculation of the FEV_1_/VC ratio leading to a lower prevalence of COPD than if the ERS definition is applied. The exclusion of persons with a low FEV_1_/VC ratio, implying airway obstruction, but with a FEV_1 _within two standard residuals of the predicted mean, is probably justified in a clinical setting. For epidemiological or preventive purposes this exclusion of a vast number of persons with mild COPD is more doubtful. In all three guidelines the importance of early detection and active smoking intervention is emphasized and it therefore seems prudent to also include mild disease (as in ERS and GOLD) in the definition of COPD in order to intensify the efforts of smoking cessation. The population impact of different definitions of airway obstruction has been described by Celli and coworkers [9] and our results further stress the need for a clear definition of chronic obstructive pulmonary disease for both epidemiological and clinical purposes.

In the NICE and GOLD guidelines, obstruction is defined as a FEV_1_/FVC ratio < 0.7. The ERS defines COPD as FEV_1_/(FVC or SVC) < 88% predicted value in men and < 89% of predicted value in women (i.e. > 1.64 residual standard deviation below predicted value). Since the predicted value of FEV_1_/SVC declines with age and the limit is higher for women (89% of predicted), the COPD diagnosis according to the ERS-definition, will include young females and exclude older men to a greater extent than the NICE and the GOLD definitions. This limitation of the GOLD criteria (in particular) for diagnosing COPD in elderly people has been described by Hardie and coworkers [10]. An important clinical implication of this is that the diagnosis of COPD may be delayed in women when the GOLD and NICE guidelines are used instead of the ERS recommendations. This is particularly contentious since smoking women are more susceptible to COPD [11,12]. In addition, men have a higher success rate in smoking cessation, which may indicate that more intensive effort is needed at an earlier stage in guiding women to successful smoking cessation [13,14].

The way of calculating the FEV_1_/VC ratio also substantially influences the prevalence of COPD. The use of either slow vital capacity (SVC) or forced vital capacity (FVC) is suggested in the ERS consensus statement from 1995 whereas the GOLD, the NICE and the ATS/ERS guidelines from 2004 only suggest the use of FVC. If the best value of FVC or SVC is used for the calculation of the FEV_1_/VC ratio instead of FVC the prevalence of COPD increases with 10–20% as shown in the present study. The disadvantage of using only FVC was greater in men and persons with impaired lungfuction (low VC and FEV_1_). Although this disadvantage diminished after bronchodilatation, it remained and could therefore not be neglected. The clinical implication of this is that a diagnosis of COPD may be overlooked if SVC is not performed, a risk that seems to be especially high in men with mild disease. Several papers have been published describing the differences between the FVC and the slow SVC in small groups of patients (< 100) with chronic airways obstruction [15-17] but the present study is the first using a large population dataset.

The reversibility to a bronchodilator (salbutamol) in the COPD patients was somewhat different depending on how COPD was defined. The NICE guidelines require a FEV_1 _below 80% of predicted value which reduces the number of positive diagnosis but, as these patients have a lower pre-bronchodilator FEV_1_, leave a greater space for increase following inhalation of a bronchodilator.

## Conclusion

Uniform international standards for the diagnosis of COPD are lacking. The existing major consensus statements and guidelines, regarding the diagnosis of COPD, yield differences in prevalence rates, which perhaps reflect that the different guidelines may primarily be intended for either clinical use or for screening and prevention. This complicates the organization of appropriate epidemiological surveys and comparisons between countries. Our results indicate that both the FVC and the SVC manoeuvre should be performed when persons at risk for COPD are examined. We also fully agree with the generally accepted concept to diagnose COPD based on lung function measurements after bronchodilatation.

## Abbreviations

ERS: European Respiratory Society;

NICE: National Institute for Clinical Excellence;

NHLBI: National Heart, Lung, and Blood Institute;

WHO: World Health Organization;

GOLD: Global Initiative for Chronic Obstructive Lung Disease;

FVC: Forced Vital Capacity;

SVC: Slow Vital Capacity;

AGS: Collective Bargaining Group Sickness Insurance (in Swedish: Avtalsgruppsjukförsäkring);

ATS: American Thoracic Society.

## Competing interests

With regard to the content of this paper none of the authors have competing interests. LN is today (but was not at the time of the study) employed as medical advicer at Boehringer Ingelheim. The study has no therapeutic implications.

## Authors' contributions

LN, MN and PM designed the study. All authors have participated in the analyses of data. LN and KL had the major responsibility for drafting the manuscript.
